# DEVELOPMENT AND EVALUATION OF AN INTERPROFESSIONAL LEADERSHIP IN HEALTH CARE CERTIFICATE

**DOI:** 10.1093/geroni/igad104.1484

**Published:** 2023-12-21

**Authors:** Bronwyn Keefe, Craig Slater, Karen Jacobs

**Affiliations:** Boston University, Boston, Massachusetts, United States; Boston University, Boston, Massachusetts, United States; Boston University, Boston, Massachusetts, United States

## Abstract

Leaders of healthcare teams have a critical role in coordinating services to deliver high-quality care to the patients and communities they serve. Leading teams can be challenging in the context of complex healthcare systems, increasing costs, resource limitations, and workforce issues. We created the Interprofessional Leadership in Healthcare Certificate for health professionals who lead, or aspire to lead, interprofessional teams. Learners complete courses relating to interprofessional collaboration, effective communication, mentoring and supervision, business acumen, and contemporary leadership models. The live classroom sessions use Project ECHO® to facilitate technology-enabled, collaborative learning. Participants completed an evaluation survey that included questions about course learning objectives, engagement in learning activities, application of content to professional practice, and interprofessional learning. The survey included open-ended questions about the most helpful aspects of each course and suggestions for changes. Evaluation data for each of the courses were collated across four cohorts. Participants represented over 10 healthcare professions. There was over 90% agreement that content met all of the learning objectives for each course. There was over 90% agreement that participants learned from other professions and engaged in interprofessional collaboration. The qualitative data indicated that learners found that the content helped them to be more intentional with their collaborative and leadership practices in their workplaces. The certificate program had a positive impact on participants’ knowledge, skills, and workplace practices relating to interprofessional collaboration and leadership. This may be attributable to intentional interprofessional collaboration in both program development and learning experiences and the use of the project ECHO model.

